# Psoriatic arthritis with skin lesions localized to the scalp: A case report

**DOI:** 10.1002/jgf2.358

**Published:** 2020-07-08

**Authors:** Tetsuya Akaishi, Kenshi Yamasaki, Yu Mori, Toshiya Takahashi, Takuya Izumiyama, Hitoshi Terui, Michiaki Abe, Shin Takayama, Setsuya Aiba, Tadashi Ishii

**Affiliations:** ^1^ Department of Education and Support for Regional Medicine Tohoku University Hospital Sendai Japan; ^2^ Department of Dermatology Tohoku University Graduate School of Medicine Sendai Japan; ^3^ Department of Orthopedic Surgery Tohoku University Graduate School of Medicine Sendai Japan

**Keywords:** apremilast, dactylitis, polyarthritis, scalp psoriasis

## Abstract

A 66‐year‐old man with a 2‐year history of suspected scalp eczema with excessive dandruff developed painful swollen joints in the extremities. Four months after developing polyarthritis and polydactylitis, eczema gradually spread to the face. He was referred to our hospital for intractable scalp and facial eczema and polyarthritis. Based on the appearance of the head and facial skin lesions, psoriasis was suspected. Treatment with apremilast (a phosphodiesterase‐4‐inhibitor) was initiated, which swiftly alleviated the skin lesions. The joint deformities persisted, but the pain in the joints disappeared. This case implies that psoriatic arthritis should be suspected even if psoriatic skin lesions are localized to the scalp.

## Graphical abstract

Psoriatic arthritis was observed in a middle‐aged man with the skin lesions localized to scalp area. Because a delayed diagnosis may result in irreversible joint deformity, close collaboration with dermatologists is essential for early diagnosis of psoriatic arthritis in patients with localized skin lesions remote from the site of arthritis or dactylitis.

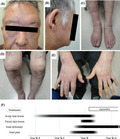

## 1 INTRODUCTION

Polyarthritis and dactylitis are among the most common complications observed in patients with psoriasis. Some patients with psoriasis show localized skin lesions on the scalp, known as scalp psoriasis. In this report, we present the case of a middle‐aged man with scalp psoriasis, who later developed polyarthritis and dactylitis without skin lesions other than scalp area.

## 2 CASE REPORT

**FIGURE 1:**
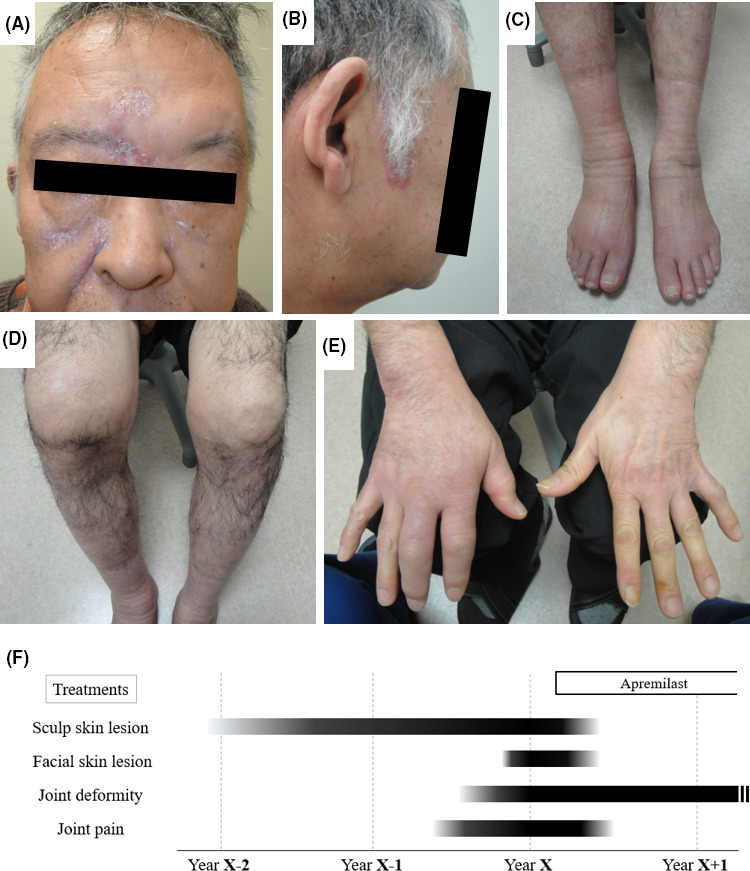
A‐E, Photographs of the patient at the first hospital visit before initiating treatment. F, Clinical course of the presented case. Polyarthritis appeared more than 1 y after the advent of the scalp lesions

**FIGURE 2:**
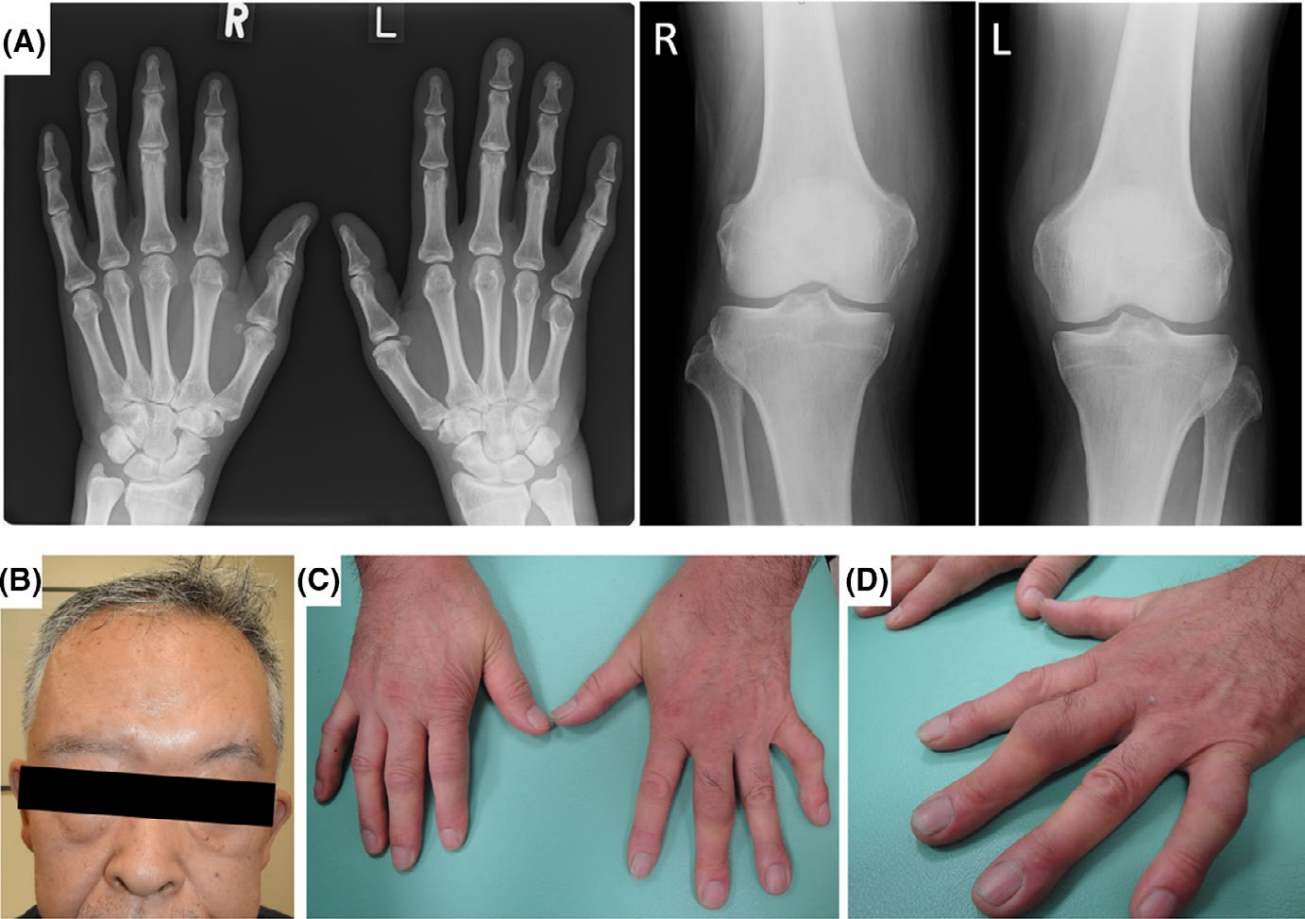
A, Hand and knee X‐rays before initiating treatment. B‐D, Six months after initiating apremilast, the scalp and face skin lesions completely disappeared. The finger joint pains also swiftly disappeared, but the joint deformity persisted

A 66‐year‐old Japanese man with a large amount of dandruff who was suspected to have scalp eczema and had been treated by a general practitioner with an anti‐inflammatory ointment for 2 years gradually developed painful swollen joints in his right hand, right knee, and bilateral ankle joints. He experienced difficulties in walking because of the severe joint pain in his legs. Because he tested negative for serum rheumatoid factor (RF), he was given a presumptive diagnosis of gout and was treated with nonsteroidal anti‐inflammatory drugs, which partially alleviated his joint pain. About 4 months after the development of polyarthritis and dactylitis, the scalp skin lesions gradually spread to his face. He remained free from any skin lesions besides those on the face and scalp. At this stage, the patient was referred to our hospital for intractable arthritis and dactylitis with joint deformities. The manifested skin lesions and arthritis together with the clinical course of this patient is shown in Figure 1. A massive amount of dandruff was observed across the scalp. The hairline of the sideburns showed reddish borders. The right knee and ankle joint were swollen and painful. Multiple sites of dactylitis with the appearance of “sausage digit” were observed in both hands, but skin lesions or nail involvement was absent. Hematological examination revealed the following findings: white blood cell count = 9300/μL (Neut: 74.3%, Eosi: 0.9%, Baso: 0.2%, Lymp: 17.8%, Mono: 6.8%); antinuclear antigen = negative; anti‐SS‐A/SS‐B antibody = negative; c‐ANCA/p‐ANCA = negative; RF = negative; matrix metalloproteinase‐3 = 324.4 ng/mL; C3 = 156 mg/dL; C4 = 48.2 mg/dL; CH50 = 81.9 mg/dL; and C‐reactive protein = 4.59 mg/dL. His liver and kidney functions were normal. In hand X‐ray images, distal phalanges of the 3rd and 4th fingers in left hand presented proliferative bony changes (Figure 2A). Scaphoid bone erosion was also suggested in the right hand. As he had already begun developing facial skin lesions, psoriatic arthritis (PsA) was suspected, and he was diagnosed with PsA by dermatologists. The patient fulfilled four of the five CASPAR criteria in 2006, except for the criterion of nail dystrophy ^1^. About 1 month after the first hospital visit, apremilast (a phosphodiesterase‐4‐inhibitor) was administered, which swiftly alleviated his scalp and facial skin lesions (Figure 2B). In addition, joint pains in his hand and legs also disappeared (Figure 2C, 2D), and he could walk without difficulties.

## 3 DISCUSSION

Psoriasis is a chronic inflammatory skin disease with genetic and environmental predisposing factors. Most patients with the disease develop well‐demarcated erythematous plaques with overlying silvery‐white scales, typically involving the scalp, trunk, buttocks, and extensor surfaces of the extremities.^2^ However, skin lesions can appear at any site on the body in patients with disease exacerbation. Both rheumatoid arthritis and PsA can cause destructive arthritis. In contrast to rheumatoid arthritis, PsA typically presents with enthesitis (inflammation of the entheses [sites at which tendons or ligaments are inserted into bones]). The prevalence of psoriasis differs between countries, and it is expected to be 0.3%‐1.2% in Japan, which is much lower than that in Western countries.^3^ The prevalence of the disease in Japan is thought to be increasing because of many possible environmental factors, including the spread of a Western‐style diet, that could also result in obesity.^4^, ^5^ Patients with psoriasis often experience systemic symptoms other than skin lesions, including arthritis, dactylitis, uveitis, and recurrent fever.^6^ Thus, not only dermatologists, but also primary care doctors are more familiar with the basic knowledge of the disease.

PsA is one of the most common complications associated with psoriasis and occurs in 5%‐30% of patients.^7^, ^8^, ^9^ Many patients with PsA present with skin lesions affecting the hands with fingernail involvement represented by nail dystrophy; however, the severity of skin lesions does not always correlate with the occurrence or severity of PsA at a specific time point.^10^ Reportedly, several features, such as scalp lesions, nail dystrophy, and intergluteal/perianal lesions, increase the risk of PsA in patients with psoriasis.^9^ Among these features of psoriasis, scalp lesions are most significantly associated with the risk of PsA. As presented in this report, severe arthritis in the fingers and extremities can be observed in patients with psoriasis whose skin lesions are localized to scalp. The importance of searching for skin lesions across the body upon evaluating patients with polyarthritis cannot be overemphasized.

Topical treatments for psoriasis include steroid ointment and vitamin D analogs. Systemic treatments for psoriasis include ultraviolet B radiation; oral administration of cyclosporin, retinoids, or apremilast; and injection of monoclonal antibodies. Patients with PsA are often treated with nonsteroidal anti‐inflammatory drugs (NSAIDs), oral disease‐modifying antirheumatic drugs (eg, methotrexate), or the injection of monoclonal antibodies (eg, tumor necrosis factor inhibitors). Oral administration of apremilast is also a useful therapeutic strategy in these patients.^11^ Axial involvement, represented by axial spondyloarthritis or active enthesitis, usually requires further treatments with monoclonal antibody therapies after NSAIDs administration; therefore, careful physical examination is important including axial joint evaluation (spine and sacroiliac joints) and/or imaging studies to detect active enthesitis.^12^ Joint deformities may persist over long periods despite initiation of appropriate therapy for psoriasis; therefore, prompt treatment is essential to avoid the onset of irreversible joint deformities.

This case highlights the importance of careful examination including scalp to diagnose psoriatic arthritis and dactylitis. Close collaboration with dermatologists is essential for early diagnosis and prompt management of PsA in patients with localized skin lesions.

## 4 ACKNOWLEDGEMENTS

None.

## 5 CONFLICT OF INTEREST

The authors have stated explicitly that there are no conflicts of interest in connection with this article.

## 6 ETHICS APPROVAL

Written informed consent was obtained to publish this case report.
